# The UNICEF/Washington Group Child Functioning Module—Accuracy, Inter-Rater Reliability and Cut-Off Level for Disability Disaggregation of Fiji’s Education Management Information System

**DOI:** 10.3390/ijerph16050806

**Published:** 2019-03-05

**Authors:** Beth Sprunt, Barbara McPake, Manjula Marella

**Affiliations:** Nossal Institute for Global Health, The University of Melbourne, Melbourne 3000, Australia; barbara.mcpake@unimelb.edu.au (B.M.); marella.m@unimelb.edu.au (M.M.)

**Keywords:** UNICEF/Washington Group Child Functioning Module, disability disaggregation, education management information system, validation, Fiji

## Abstract

This paper explores the validity (sensitivity and specificity) of different cut-off levels of the UNICEF/Washington Group Child Functioning Module (CFM) and the inter-rater reliability between teachers and parents as proxy respondents, for disaggregating Fiji’s education management information system (EMIS) by disability. The method used was a cross-sectional diagnostic accuracy study comparing CFM items to standard clinical assessments for 472 primary school aged students in Fiji. Whilst previous domain-specific results showed “good” to “excellent” accuracy of the CFM domains seeing, hearing, walking and speaking, newer analysis shows only “fair” to “poor” accuracy of the cognitive domains (learning, remembering and focusing attention) and “fair” of the overall CFM (area under the Receiver Operating Characteristic curve: 0.763 parent responses, 0.786 teacher responses). Severe impairments are reported relatively evenly across CFM response categories “some difficulty”, “a lot of difficulty” and “cannot do at all”. Most moderate impairments are reported as “some difficulty”. The CFM provides a core component of data required for disaggregating Fiji’s EMIS by disability. However, choice of cut-off level and mixture of impairment severity reported across response categories are challenges. The CFM alone is not accurate enough to determine funding eligibility. For identifying children with disabilities, the CFM should be part of a broader data collection including learning and support needs data and undertaking eligibility verification visits.

## 1. Introduction

It is critical that education data systems are disaggregated by disability to measure progress in achieving access to quality education for children with disabilities, and efforts to enable this are moving forward globally. Disability-disaggregated education data are required to track progress towards various frameworks including the Convention on the Rights of Persons with Disabilities (CRPD) [1], the Sustainable Development Goals (SDG) [2] and the Incheon Strategy to “Make the Right Real” for Persons with Disabilities in Asia and the Pacific [3]. There is widespread consensus on the urgency to support Ministries of Education (MoEs) to disaggregate their Education Management Information Systems (EMISs) by disability, and the importance of doing so using tools which are valid and internationally comparable [2,4,5]. Given the complexity of disability measurement, efforts to develop and agree upon tools for disability measurement that are valid, feasible and comparable have taken statisticians and researchers decades. Whilst debate remains lively, the urgency to gather baseline data for the SDGs has required consensus. In a statement titled *Disability data disaggregation joint statement by the disability sector* [6], peak disability agencies such as the International Disability Alliance, the World Health Organization, UNICEF, United Nations Development Programme, and the UN Partnership to Promote the Rights of Persons with Disabilities, amongst others, agreed that the Washington Group on Disability Statistics (WG) modules should be used to disaggregate data sets to measure SDG indicators; the WG Short Set of questions for adults and the UNICEF/WG Child Functioning Module (CFM) for children.

The CFM has been developed for measuring child functioning in surveys with parents/caregivers as proxy respondents for the child’s functioning. It has been validated in different settings [7,8,9,10,11,12,13,14,15,16] and was finalised in 2016. The CFM is designed for children between two and 17 years and covers a range of areas for measuring functioning difficulties, including: seeing, hearing, walking, self-care, speaking, learning, remembering, anxiety/worry, depression/sadness, controlling behaviour, attention/concentrating, accepting changes in routine and making friends. Response categories for most questions are: “no difficulty”, “some difficulty”, “a lot of difficulty” and “cannot do it at all”.

Recent advice from the United States Agency for International Development (USAID), a key donor, is that the WG Short Set and/or the CFM should be used wherever possible in USAID-funded education programs to disaggregate data sets [17]. This is a positive indication of donor commitment to measuring outcomes towards fundamental human rights. If the CFM is to be used to disaggregate EMISs, it is critical that its properties are understood when proxy respondents are teachers and to test its measurement accuracy when used for education systems.

There are various purposes for which disability identification is needed in data aimed at ensuring inclusion of children with disabilities. From determining funding eligibility at an organizational or an individual level, determining learning and support needs of a student, to comparing equalization of access to socioeconomic rights through disability-disaggregated census or household survey data. The purpose has implications for the approach to disability identification, and for the degree of accuracy required in the instrument that determines the classification of disability. Madden highlighted the importance of designing valid tools which take into account the evidence for and consequences of score interpretation and use, and establishing meaningful thresholds on the spectrum of disability experience [18].

Disability can be seen as a continuum ranging from minimal difficulties to fundamental impacts on a person’s life. On an instrument designed to measure this functioning to disability continuum, the point along the span which is used to define someone as having a disability is referred to in this paper as a cut-off. It is critical that the rationale and implications of the cut-off are clearly understood. If for example the cut-off is relatively low on the continuum and includes mild disabilities (such as difficulty seeing which can be entirely overcome with glasses), the number of children counted as having disability will be high. Whereas if severe disability is the cut-off (having a great deal of difficulty with basic functions), the number of children counted as having disability will be comparatively low. The cut-off level must be appropriate to, and will alter depending on, the purpose for identifying disability. Education systems may consider it important to identify children with mild and moderate disability to enable early intervention and educational accommodations, whereas a scheme establishing eligibility for monetary benefits may target a higher level of disability [19].

The recommended criterion for identifying disability using CFM is having difficulty functioning at the level of at least “a lot of difficulty” [20], or “daily” for anxiety and depression questions. The USAID guidance document [17] states that “*for a more nuanced analysis of disability, the answers can be used as a regular scale, with “cannot do it at all” denoting severe disability while “some difficulty” denoting minor disability in each functional domain. Answers across all domains can also be combined into a larger scale.*” (p. 4). However recent studies in Cameroon, India and Fiji [12,21,22,23] indicate that there is a significant variation in how parents choose response categories to report functioning difficulties and that the cut-off “a lot of difficulty” misses significant numbers of children with moderate to severe impairments. That is, this cut-off had low sensitivity in identifying disability. When used in large household surveys or censuses the importance of these differences may be considered within acceptable margins of error. However, within an education system the tool is used for different purposes and a response cut-off with a high sensitivity is needed. Sensitivity and specificity are a trade-off and selecting a lower severity response category, for example “some difficulty”, may result in lower specificity. That is, the chance increases of falsely identifying some children as disabled who do not have a disability.

In a rapidly modernising information technology age, EMISs are increasingly based on individual electronic data files [24]. Data from these systems are not only used to monitor and evaluate progress towards inclusive education at a large area level but are capable of and being used to determine individual student eligibility for funding related to disability status. A tool appropriate for national surveys may not also be reliable or valid in identifying individual students’ levels of functioning. It is critical that people making decisions about incorporating disability within EMISs understand that tools they are being advised to use for national or large area monitoring may have limitations for individual level assessments.

This study was undertaken in the context of an Australian aid funded education sector project in Fiji. The required purposes for disability data in Fiji’s EMIS included identification of children with disabilities, by disability type and severity, to enable resource allocation based on individual level data, and to enable monitoring, planning and reporting against policy and other commitments. The key question for the Fiji MoE was the extent to which the CFM is effective when used by teachers to identify the presence and severity of disability amongst children in Fiji. Validity and reliability of specific domains (seeing, hearing, walking, speech and cognition) were reported elsewhere [21,22,23]. This paper focuses on the performance of the CFM as a whole. With the overarching aim of identifying a valid, reliable and feasible method for Fiji to identify children with disabilities in schools to enable monitoring, planning and reporting against policy commitments, the objectives of this paper are to:

(1) Determine the validity (sensitivity and specificity) of different cut-off levels of the CFM for predicting the presence of disabilities in primary school aged Fijian children compared to standard clinical assessments of impairment.
(2) Determine the inter-rater reliability between teacher and parent CFM responses.

## 2. Materials and Methods

### 2.1. Study Design and Sampling

A cross-sectional diagnostic accuracy study, two-gate design with representative sampling [25] was undertaken from March-July 2015 in Fiji. In diagnostic accuracy studies, the index test whose accuracy is being investigated (CFM) is compared to reference standard (clinical) tests, sometimes termed “gold standards” [26,27]. The purpose of a diagnostic accuracy study is to evaluate the ability of the index test to correctly classify study participants into two categories, those with and without the ‘target condition’. Diagnostic accuracy is based on measuring sensitivity and specificity values at each cut-off level. For the purpose of assessing the sensitivity and specificity of the CFM against the reference tests, we have essentially defined disability as clinically assessed impairment of a moderate or more severe level. There are inherent limitations in assuming that medical impairment assessments are “gold standards” for disability. However, this approach enabled a validated, consistent and objective means of measuring an aspect of disability, i.e., impairment, against which the self-report-based CFM could be compared.

Ethics approvals were obtained from the University of Melbourne’s Human Research Ethics Committee (#1543942, 17/03/15) and the Fiji MoE’s ethics committee (RA09/15, 5/03/15). All subjects had written consent and children’s assent was obtained prior to each clinical assessment. Sampling was purposive regarding school selection and student participation. Participants for the study were 5–15 year old students recruited from ten special schools and five inclusive education (mainstream) schools from the four administrative divisions in Fiji. Children invited to participate included: all children in the special schools, and all children in the mainstream schools previously identified by the school to have disabilities, and selected controls matched by age, sex, ethnicity and location (Table 1). The flowchart of participation is shown in Figure S1 (Supplementary Material). Invitations to parents were included in the information and consent process for participation of the children. Teachers in all study schools were informed of the research and given information and consent forms. After the children had been assessed and parents interviewed, respective teachers of the children were provided questionnaires to complete. Representative sampling focused on including cases with mild/moderate through to profound impairment to minimise “spectrum effect”, whereby a sampling bias towards including only cases with more significant impairment can lead to higher estimates of sensitivity and specificity [25]. This was operationalized in two ways: (i) by keeping tallies on impairment levels of children throughout recruitment and working closely with schools to achieve a mixture of impairment severity levels; and (ii) by assessing large numbers of children who were not initially identified by schools as having disability, which resulted in a sample with a full spectrum of function/impairment, including those around the lower or borderline end, which was necessary to minimise “spectrum effect”. Sample size was estimated based on minimum number to achieve a sensitivity or specificity of 0.85 (prevalence 0.10, alpha 5%, 1-beta 80%; CI 95%, lower confidence limit 0.65) [28]. A target of 52 cases and 52 controls were sought under each of five impairment domains (vision, hearing, musculoskeletal, speech and cognition).

### 2.2. Test Methods

#### 2.2.1. Index Test—Child Functioning Module

This study used a draft of the CFM (5–17 year age group) current at February 2015, with permission from UNICEF and the Washington Group. Appendix A lists the differences between the version used in the study and the final version of the CFM, which is available from www.washingtongroup-disability.com. Translation and pretesting processes are described in [21]. For the diagnostic accuracy analysis in this paper, only seven CFM domains are included (seeing, hearing, walking, speaking, and three cognitive domains—learning, remembering and focusing attention) as these relate directly to constructs measured in the clinical assessments.

For clarity, the term “CFM-7” is used throughout this paper when referring to this group of domains. For other analysis in the paper the remaining domains (self-care, anxiety/worry, sadness/depression, controlling behaviour, accepting changes to routine and making friends) are included and the term “CFM-13” is used to refer to the entire module. Table 2 provides the wording of the CFM questions and response categories and illustrates the domains referred to by the terms CFM-7 and CFM-13.

#### 2.2.2. Reference Standard (Clinical) Tests

Clinical tests were undertaken for vision, hearing, musculoskeletal impairment, speech and cognition using reference standard (clinical) tests considered the best available tests regarding the conditions of interest [26,27]. The clinical tests for this study were selected based on international standards for vision and hearing and well validated tools for speech, musculoskeletal impairment and cognitive impairment. Detailed descriptions of these assessments and how they were implemented in this study are available elsewhere [22,23,24] and summarised in Appendix B.

Case definitions. Vision impairment: presenting visual acuity in the better eye <6/18 and ≥6/60 (moderate), <6/60 and ≥3/60 (severe) and <3/60 (blind) [29]. Hearing loss: 41–60 dBA (moderate), 61–80 dBA (severe) and ≥81 dBA (profound). Children identified on the Rapid Assessment of Musculoskeletal Impairment with structure impairment including “severe”, “moderate” and “mild” effect on the musculoskeletal system’s ability to function as a whole were identified as cases with mobility impairment [30]. Children identified to have impairment only affecting the upper limb were excluded to enable comparison with the CFM question on walking. Speech impairment: Intelligibility in Context Scale [23,31] scores: 1.8 to <2.5 (moderate) and 1.0 to <1.8 (severe). Cognitive impairment: assessed using the Cambridge Neuropsychological Test Automated Battery (CANTAB) [32], subjects with Overall Impairment Scores of 3 (moderate) and 4–5 (severe) [22].

#### 2.2.3. Implementation of the Index Test and Clinical Tests

Assessment camps were run over two to five days at each school in rooms set up with multiple assessment stations. Parents/caregivers attended the screening camp where an interviewer administered the CFM in a location separate from the reference standard assessments, using either the Fijian, Fijian-Hindi or English version depending on parent preference. Interviewers had received a half-day training in administration of the questionnaire. In-situ training also occurred during the early stages of data collection, with the lead researcher providing clarification about administration as questions arose. It was self-completed by teachers either during the camp or within the following week; teachers received no training other than instructions to carefully follow the skip-prompts in the questionnaire. The clinical team were blinded to the CFM results and teachers and parents were blinded to each other’s CFM responses and to clinical results.

### 2.3. Data Analysis

Statistical analysis was undertaken using SPSS Version 24 (IBM, Armonk, NY, USA) and MedCalc v.17.6 (MedCalc Software, Ostend, Belgium). Descriptive statistics were calculated for participant demographics and CFM-7 results were cross-tabulated by clinical results. To analyse diagnostic accuracy of the CFM-7, the case definition was: child has impairment in at least one of the five clinical assessments (see “Case definitions” above). The definition to determine CFM-7 response was the highest level of difficulty reported against any of the seven domains. For example, for a child assessed as having “a lot of difficulty” seeing and “some difficulty” speaking, the overall CFM response would be recorded as “a lot of difficulty”.

Sensitivity (Sn), specificity (Sp) and likelihood ratios (LR) were calculated for each respondent type (parent or teacher) for each cut-off level. True positives are children with impairments (assessed using the reference standard (clinical) assessments, defined by the case definitions in Section 2.2.2), who are correctly identified by the CFM as having difficulty in the respective functioning domain. True negatives are children without impairments who are correctly identified by the CFM as not having difficulty in the respective domain. False positives are children without impairments who are incorrectly identified by the CFM as having difficulty. False negatives are children with impairments who are incorrectly identified by the CFM as not having difficulty. Positive (and negative) LRs indicate how many times more likely a positive (or negative) test result is obtained when the target condition is present than when it is absent:

Sn = true positives/total cases
Sp = true negatives/total controls
Positive LR = Sn/(false positives/total controls)
Negative LR = (false negatives/total cases)/Sp

Receiver operating characteristic (ROC) curves were constructed separately for parent and teacher CFM-7 responses to determine the Area Under the ROC Curve (AUC). ROC curves are constructed by plotting the false-positive rate (1—specificity) against the true-positive rate (sensitivity) at each cut-off value defined by the CFM and then drawing a line from x = 0, y = 0 through the values at each cut-off point; the AUC is an overall figure of diagnostic accuracy with a perfect test having a value of 1.0 and a value of 0.5 suggesting that the test result is no better than chance [33,34]. AUC interpretations were classified as excellent (0.96–1.0), very good (0.9 to <0.96), good (0.8 to <0.9), fair (0.7 to <0.8), poor (0.6 to <0.7), and useless (0.5 to <0.6) [33]. ROC curves used dichotomous clinical variables, differentiating cases and controls based on definitions outlined earlier.

The Youden Index (YI) was calculated for each ROC curve to determine the statistically “optimal” cut-off level for each disability domain (seeing, hearing, walking, speaking, learning, remembering and focusing attention) and respondent type. The YI is the maximum vertical distance between the ROC curve and the line of random chance ([x = 0, y = 0] to [x = 1, y = 1]) and is calculated as maximum (Sn + Sp − 1). That is, the cut-off point at which (sensitivity + specificity − 1) is maximal, is taken to be the “optimal” cut-off point [35]. Importantly, the YI gives equal weight to false positive and false negative values, which means that it does not vary based on the context or aim of the test. For the purpose of this study, it is a useful index to provide consistency in our comparisons between disability domains, the CFM as a whole, and respondent types. For determining the best choice, or contextually “optimal”, cut-off level for Fiji’s MoE, the advantages and disadvantages of valuing sensitivity or specificity more highly are considered in depth in the Discussion.

Throughout the paper, results related to parents as proxy respondents are denoted by a subscript P and those by teachers by a subscript T.

For the domains without clinical reference standards in this study (self-care, anxiety, depression, controlling behavior, accepting changes to routine, and making friends), proportions of the sample reported as ≥ “some difficulty” and ≥ “a lot of difficulty” were compared. These two cut-off values were compared because the recommendation from the WG is to use “a lot of difficulty” [20,36] but previous results have raised concerns about the low sensitivity of this cut-off [13,21,23]. Also, a comparison of the clinical impairments of children identified at both cut-offs was undertaken, comparing “some difficulty” to ≥ “a lot of difficulty” on the CFM-13.

Inter-rater reliability between parents and teachers was tested using a two-way random, absolute, average-measures intra-class correlation (ICC) [37]). Using Cicchetti’s classification [38], IRR interpretations were classified as: poor (<0.40), fair (0.40–0.59), good (0.60–0.74), and excellent (0.75–1.00). Cicchetti is slightly more generous than other classifications [39,40].

Spearman’s rho correlation coefficient was used to test correlations between age, gender, and school type and CFM items, using the criteria: very high (0.90–1.00), high (0.70 < 0.90), moderate (0.50 < 0.70), low (0.30 < 0.50) and negligible (0.00 < 0.30) [41].

Unless otherwise noted the two CFM questions on difficulty being understood when speaking by people: (1) inside the household, and (2) outside the household, have been combined as per the WG recommendation - to use the most severe response reported for either question [20].

## 3. Results

### 3.1. Participant Demographics and Distribution of Impairments

The sample included 472 children with mean ± SD age of 10.2 ± 2.6 years (range: 5 to 15 years) in Classes 1 to 8, including approximately half from special and half from mainstream schools (Table 1). There were 231 cases in the study and 241 controls, determined by clinical assessments. Cases included 35 children with vision impairment ranging from moderate vision impairment to total blindness, 60 children with hearing impairment from moderate hearing loss to profound deafness, 42 children with mild to severe mobility impairments, 71 children with moderate to severe speech impairment, and 125 children with moderate to severe cognitive impairment (Table S1). The mean age of cases was 10.15 years and controls was 9.71 years. Females made up 37.2% of cases and 51.0% of controls. Ninety-eight teachers participated, of whom 69% were female. Of the parents/guardians of the cases: 56% were mothers, 19% fathers, and 25% other (grandparent, aunty, uncle, guardian); the highest level of education was primary for 25%, secondary for 56% and higher education for 19%. Of the parents/guardians of the controls: 60% were mothers, 25% fathers, and 15% other; the highest level of education was primary for 22%, secondary for 63% and higher education for 15%.

### 3.2. Validity (Sensitivity and Specificity) of Different Cut-Off Levels of the CFM

#### 3.2.1. Diagnostic Accuracy of the CFM

Table S2 presents values of area under the curve (AUC), sensitivity, specificity, the Youden Index for the optimal cut-off points and likelihood ratios from the construction of ROC curves. Table 3 provides a summary of key data from Table S2.

Domain-specific results shown in Table 3 (eg., seeing, hearing) are discussed elsewhere and provided here to enable comparison with the overall CFM-7 result (see Table 2 for definition of CFM-7). In summary, the accuracy (AUC) of the CFM items on seeing, hearing, walking and speaking were higher than the items on learning, remembering and focusing attention. The lower levels for learning, remembering and focusing attention led to the CFM-7 as a whole having an AUC that was only “fair” (0.763_P_/ 0.786_T_); with slightly better overall accuracy by teachers. As shown in Table S2, levels of sensitivity were very consistent between parents and teachers across the cut-off levels, with “some difficulty” being 0.98_P_/0.96_T_, “a lot of difficulty” being 0.55_P_/0.57_T_, and “cannot do at all” being 0.23_P_/0.22_T_. Whilst teachers had higher specificity than parents at the cut-off “some difficulty” (0.33_P_/0.42_T_), results were more consistent at the higher levels; “a lot of difficulty” being 0.80_P_/0.82_T_, and “cannot do at all” being 0.99_P_/0.99_T_.

#### 3.2.2. Cross-Tabulation of CFM Results by Clinical Test Results

Table 4 presents the spread of CFM-7 responses across impairment levels - none, mild, moderate and severe. Table S3 provides an extended presentation of Table 4 showing cross-tabulation of the highest level of severity of the child on any of the five reference standard results (vision, hearing, musculoskeletal, speech, cognition) with the highest level of difficulty reported for that child on any CFM-7 response.

There was strong consistency between parent and teacher results in the overall proportions of children categorised as having “a lot of difficulty” (25.8%_P_/26.5%_T_) and “cannot do at all” (11.4%_P_/12.5%_T_). Parents reported slightly more children as having “some difficulty” (44.9_P_/39.3%_T_) and slightly fewer children as having “no difficulty” (17.6%_P_/21.7%_T_). Most moderate impairments are reported by parents and teachers as “some difficulty”. Severe impairments are reported approximately evenly across three CFM response categories: “some difficulty”, “a lot of difficulty” and “cannot do at all”. Most children with no impairments are mainly reported as having “no difficulty” (33.9%_P_/43.8%_T_), or “some difficulty” (47.4%_P_/39.1%_T_). However, a notable proportion (17.8%_P_/16.0%_T_) are reported as having “a lot of difficulty”, which is predominantly related to items on learning, remembering and focusing attention (as shown in Table S3). Children with mild impairments are mainly reported as having “some difficulty” (42.1%_P_/58.8%_T_) and “a lot of difficulty” (47.4%_P_/29.4%_T_).

Problematically, the response category “some difficulty” includes children with a wide range of functioning. Of children with moderate clinical impairments, 52.4%_P_/47.3%_T_ are reported as just having “some difficulty”, and of the children with severe impairments, 38.8%_P_/34.4%_T_ are recorded as just “some difficulty”.

#### 3.2.3. ROC Curve Analysis Implications for Cut-Off Level

Table 3 (and Table S2) show the YI for parent and teacher responses at the cut-off levels “some difficulty” and “a lot of difficulty” for each domain-specific question and for the CFM-7. For all seven domain-specific questions, the YI for the cut-off “some difficulty”, for both parent and teacher responses, is clearly higher than the YI for the cut-off “a lot of difficulty”. However, when considering the accuracy results for the CFM-7 (that is, the combined results), this is reversed and the cut-off “a lot of difficulty” is the highest.

The positive likelihood ratio at the level of “some difficulty” is 1.46_P_/1.66_T_, compared to 2.78_P_/ 3.21_T_ at the level “a lot of difficulty”. This means that the cut-off “some difficulty” provides a ‘minimal increase’ in the probability of the CFM-7 identifying disability in a child with disability compared to a child without. This is improved upon only somewhat by the cut-off “a lot of difficulty” which provides a ‘small increase’. The negative likelihood ratios for the overall CFM-7 at the cut-off “some difficulty” indicate a ‘large and often conclusive’ decrease in the likelihood that a negative result comes from a child with disability than from a child without disability. Whereas at the cut-off “a lot of difficulty” there is only a ‘small’ to ‘minimal decrease’ in this likelihood. These results should be interpreted cautiously though because the confidence intervals for the higher cut-offs were very wide due to small sample sizes.

#### 3.2.4. Domains without Clinical Reference Standard

Table 5 summarizes the analysis of CFM domains that did not have clinical reference standard tests—self-care, anxiety, depression, controlling behaviour, accepting changes, and making friends. It highlights the proportion of responses for each domain at the level of at least “some difficulty” compared to at least “a lot of difficulty”. Parents and teachers reported a similar proportion having at least “some difficulty” with self-care (20.1%_P_/21.6%_T_), with “good” correlation between respondents (0.72). However, teachers reported a higher proportion having at least “a lot of difficulty” with self-care (2.3%_P_/6.2%_T_). Parents and teachers reported a similar proportion of the sample as feeling anxious or depressed “weekly”, but correlation was “negligible” (≤ to 0.26). Teachers reported a higher proportion of the sample as feeling anxious or depressed “daily”. Whilst data are not shown here, teacher responses showed a high correlation between learning and remembering (0.758), and depression and anxiety (0.729), and a moderate correlation between accepting changes to routine and focusing attention (0.546), self-care and walking (0.520), learning and being understood outside (0.511), focusing attention and learning (0.502), and accepting changes to routine and learning (0.502). Parent correlations for the same domains were far lower, ranging from 0.152–0.527.

Overall, the proportions of children reported as “some difficulty” in the domains in Table 5 seem very high, but without a reference standard it is not possible to know whether this is reflective of disability.

#### 3.2.5. Impairments Represented within Cut-Off Levels across the CFM-13

To further explore the rate of clinical impairments amongst children identified at the two cut-off levels (“some difficulty” and “a lot of difficulty”), Table 6 shows the frequencies of any impairment occurring amongst children reported as having “some difficulty” compared to ≥ “a lot of difficulty” on any question on the CFM-13. Table 7 is similar, but shows the frequencies of the individual impairments. As expected, with the larger number of questions on the CFM-13, slightly fewer children are missed compared to the CFM-7.

Table 7 shows that children with moderate impairments that would be missed if the cut-off were “a lot of difficulty” are spread across all types of impairments, however it is the cognitive impairments that are missed more than other impairment domains.

Using the “a lot of difficulty” cut-off, 39.7%_P_/33.3%_T_ of the children with moderate impairments and 27.5%_P_/20.5%_T_ of the children with severe impairments would be missed. Of all the types of impairment, those with moderate or severe cognitive impairment form the greatest proportion of children who would be missed if the cut-off were “a lot of difficulty”. These results do not indicate how many children with other impairments such as psychosocial or behavioural (which require other clinical assessments) may be missed.

### 3.3. Inter-Rater Reliability of the CFM

**Figure 1:**
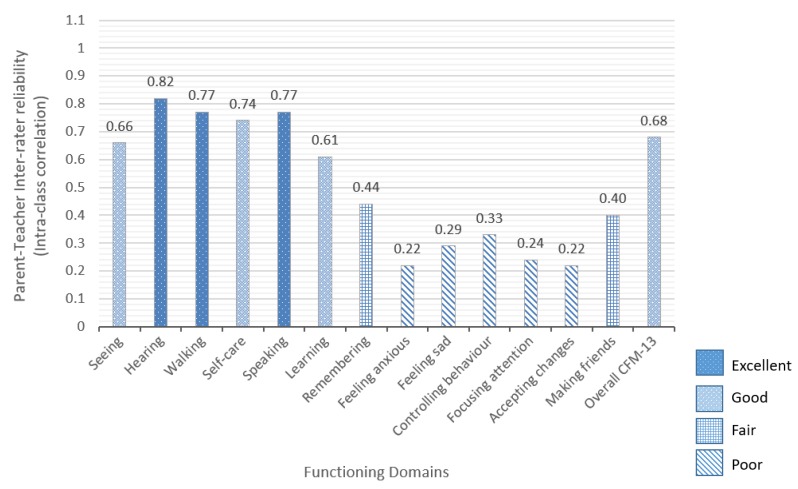
Inter-rater reliability between parents and teachers of the overall CFM (CFM-13) and of individual domains.

Inter-rater reliability between parents and teachers, assessed using ICC, varied considerably across disability domains as shown in Figure 1.

For the overall CFM-13 it was 0.68 (95% CI 0.60–.73). The range of ICC was 0.22–0.82 across the individual domains. Domains with better ICC (0.61–0.82 were hearing, walking, speaking, self-care, seeing and learning. Domains with lower ICC (0.22–0.33) were anxiety, sadness, controlling behaviour, focusing attention and accepting changes to routine. Table 6 shows better correlations for overall categorisation of children with no impairment (0.61) and mild impairment (0.85) across the categories “some difficulty” and “a lot of difficulty”. However, correlations are worse for children with moderate impairment (0.06, not significant) and severe impairment (0.55). On the whole, correlations between teachers and parents were variable.

## 4. Discussion, Limitations and Further Research

This study identified that the CFM is a useful core aspect of data required for disability disaggregation of Fiji’s EMIS and that teachers are adequately accurate proxy respondents to the CFM. However, the mixture of severity of impairments reported across CFM response categories and ambiguity in the choice of cut-off level, in both parent and teacher results, are limitations of the CFM and indicate that the CFM may not be accurate enough to be used as the sole method for identifying children with disabilities.

The first objective of this study was to determine the validity (sensitivity and specificity) of the CFM, which is operationally defined as the extent to which an overall score on the CFM at a given cut-off level identifies children who have an impairment as assessed using reference standard, or “gold standard”, clinical measures. For assessing sensitivity and specificity of the CFM, this paper effectively defines disability as clinically assessed impairment of a moderate or more severe level. There is debate about this medical perspective but for our purposes, it provides an objective assessment (in the sense of being made independently of those who stand to gain or lose from the assessment, or might perceive that they do), and so we have accepted it as the best available reference standard.

Overall diagnostic accuracy (a combined value of sensitivity and specificity) of the CFM was found to be just “fair” based on combined results from seeing, hearing, walking, speaking, learning, remembering and focusing attention, i.e., CFM-7. This is substantially lower than the previously reported accuracy of individual domain-specific questions on speaking, walking, seeing and hearing [21,23], which are perhaps more observable functions. The cognitive domains had “fair” to “poor” accuracy (22). Given the variation in accuracy across the different domains in the module ranging from excellent to poor, it is not surprising that overall accuracy is only “fair”. This finding indicates that CFM-7 may not be accurate enough to be used as the sole method for identifying children with disabilities.

Whilst diagnostic accuracy of parent observations related to seeing, walking and speaking is stronger than that of teachers, teacher accuracy is acceptable, ranging from “good” to “very good” (between 0.823–0.909). Conversely, for the domains learning, remembering and focusing attention, teacher results are stronger than parent results. For hearing, the accuracy is high and very similar between respondent types.

To disaggregate Fiji’s EMIS by disability, it is important to identify the appropriate cut-off level of the CFM. The field testing of CFM as part of population-based surveys in Samoa, Mexico and Serbia showed that the “some difficulty” cut-off estimates a very high prevalence compared to the “a lot of difficulty” cut-off [15]. The cut-off recommended by UNICEF/ Washington Group is “a lot of difficulty” [20]. However, in our study a significant proportion of children with moderate or higher clinical impairment were reported as having only “some difficulty” on CFM-7, comprising seeing, hearing, walking, speaking, learning, remembering and focusing attention domains (Table 3). These children would therefore miss out on services if the cut-off were “a lot of difficulty”. Based just on these domains, approximately half of children with moderate clinical impairments (52.4%_P_/47.3%_T_) and a third of children with severe impairments (38.8%_P_/34.4%_T_) would miss out on services if the cut-off level were “a lot of difficulty”. However, when CFM-13 was considered (which includes the additional 6 questions), not surprisingly the chance of missing children is reduced, and the proportions were reduced to some extent. Despite this, 39.7%_P_/33.3%_T_ of children with moderate clinical impairments and 27.5%_P_/20.5%_T_ of children with severe impairments would be missed. When domain-specific findings are considered, it is the children with moderate-severe cognitive impairments who miss out in greatest numbers [21,22,23]. The decision to select a cut-off must also consider the fact that 47.8%_P_/39.1%_T_ of children with no clinical impairment are reported as having “some difficulty”. Our findings indicate that children reported as having “some difficulty” can neither be ignored nor be assumed to have disability.

The cross-tabulation also highlights the fact that the three CFM response categories—“some difficulty”, “a lot of difficulty” and “cannot do at all”—do not relate to the same levels of severity across different functioning domains. This is in contrast with the recommendations on the interpretation of these categories by UNICEF/Washington Group [20] and USAID [17]. Whilst most moderate impairments are reported as “some difficulty”, children with severe impairments are showing up relatively evenly across the three response categories, and the response categories do not have the same meaning across different domains. For example, the category “cannot do at all” picks up a large proportion of children with severe musculoskeletal impairment yet it picks up only approximately 2% of children with severe cognitive impairment. This extreme response category is used to a small extent for questions on hearing, walking, speaking and seeing, but almost never used for questions on learning, remembering and focusing attention.

The CFM is described as being able “to determine the proportion of those who have mild difficulties (at least *some difficulty* on one or more domains of functioning), or moderate levels of difficulty (those who respond at least *a lot of difficulty*) or those with severe difficulties (those who respond *cannot do at all*)” [36] (p. 487). However, our findings suggest that this interpretation of the CFM response categories across disability domains would not work in Fiji. Mitra emphasised the value of using a “trichotomy” (severe, moderate and no difficulty), in which classification of people with moderate functional difficulty was based on “some difficulty” in at least one domain with no higher levels of difficulty recorded [43]. This is consistent with our finding that the cut-off “some difficulty” included most of our children with moderate impairments, however the challenge remains that many children without impairments were also recorded as having “some difficulty”.

The ROC curve results from earlier reports were complicated and varied across domains and methods, including sensitivity, specificity, the Youden Index and likelihood ratios. For the domains seeing, hearing, walking and speaking, “some difficulty” was a far more accurate cut-off than other levels [21,23]. The cognitive domains learning, remembering and focusing attention also indicate the cut-off “some difficulty” as the best, with teacher results being superior to parents at identifying children with cognitive impairments [22].

However, contrary to the individual domain-specific results, the diagnostic accuracy results for the CFM-7 showed “a lot of difficulty” as the best cut-off, albeit only marginally better. This is because at “some difficulty” sensitivity is excellent (0.98_P_/0.96_T_) but specificity is very poor (0.33_P_/0.42_T_). At the cut-off “a lot of difficulty” specificity was much better (0.80_P_/0.82_T_) but sensitivity dropped significantly (0.55_P_/0.57_T_). Notably, the Youden Index for the overall CFM was quite low at either cut-off (0.31_P_/0.40_T_ for “some difficulty” and 0.36_P_/0.39_T_ “a lot of difficulty”). This was not surprising given the disappointing diagnostic accuracy of the CFM-7 as only “fair”. These results further highlight an important shortcoming in diagnostic accuracy of the CFM-7: there is no clear and strong cut-off response category for the overall CFM and the cut-off which performs best for individual functional domains is different from that for the overall module.

The high proportion of children reported as having “some difficulty” on the six domains without a clinical reference standard highlights the need for further research to understand the impact of the cut-off level on identifying children with difficulties in these domains.

The second objective was to determine the inter-rater reliability between teacher and parent CFM responses. Our study showed that IRR of the CFM-13 is “good” (0.68), which in theory contributes to the case that the CFM can be used with teachers as respondents. However, there is great variation in IRR across domains [21,22,23]. The potentially more observable domains (hearing, walking and speaking) have “excellent” IRR followed by “good” IRR for self-care, seeing and learning.

However, IRR needs to be considered in relation to accuracy. For example, if both respondents are equally “wrong”, the IRR may be high but this does not mean the tool is useful. Or, if parent responses are “wrong”, a low IRR could be positively interpreted in terms of teacher use of the tool. Considering accuracy together with IRR between parents and teachers, the most accurate and reliable CFM questions relate to the domains of seeing, hearing, walking and speaking. Of the CFM questions for which this study does not have clinical reference standards (and therefore no diagnostic accuracy analysis)—self-care, anxiety, sadness, controlling behaviour, accepting changes and making friends—it is harder to interpret the largely poor IRR results. This may reflect poorly on the questions or may imply varying perspectives and accuracy between parents and teachers; teachers may be in a better position to make a relative judgment for some of these items. The higher correlations between teacher results for domains which might be expected (anxiety and depression; learning and remembering; changes to routine and focusing attention) provide some indication that teachers are observing these functional domains more consistently than parents and that teacher results may be more accurate in these domains. In relation to anxiety and depression, the results highlight a potentially important role for teachers in Fiji in identifying children at risk of psychosocial distress. These issues both point to important areas for future research. Research is required to investigate parent and teacher response accuracy for these domains.

Fiji’s MoE has committed to provide inclusive education in a way which leaves no one behind [44] and following this study commenced disability inclusion grants to schools, calculated by number of children with disabilities. Messick [45] and Shepard [46] championed the importance of undertaking “consequential validity”, or investigation and prediction of positive and negative social consequences of a test. The implication of Fiji’s policy, in relation to this study, is that if a cut-off level has a low sensitivity it misses out eligible children, which would be the case if “a lot of difficulty” were used. Hence to ensure children are not missed the cut-off “some difficulty” must be used. However, given the significant proportion of children classified as “some difficulty” who do not have disability, follow-up assessments are required to verify presence of disability (and to identify children for whom referral services are required).

Conversely the low specificity of the “some difficulty” cut-off has cost implications regarding verification visits. Travelling to remote areas to assess children simply based on a self-reported “some difficulty” response would be cost-prohibitive and an inefficient use of already stretched MoE staff time. A solution to this challenge may be found in another series of results from the study, to be discussed in a subsequent paper, showing that the combination of CFM data and learning and support needs data enables a much more accurate estimation of disability. This would reduce false positives on the list of children who need verification visits.

An essential feature of the CFM to highlight, in relation to assessing disability for funding eligibility, is the self-report nature of the tool. Whether the respondent is a parent/caregiver or a teacher, the results can be biased if there is perceived financial advantage in reporting higher levels of difficulty. The disability verification visit is necessary to pre-empt over-reporting. These visits involve qualified MoE district officers visiting the schools to discuss the results with teachers and undertake basic tests with the identified children, such as visual acuity tests (Snellen chart), observations of gross and fine motor function, classroom observation, review of student records, etc. The visit offers the chance for monitoring and mentoring of efforts towards disability-inclusive education.

### Limitations

An important limitation common to all diagnostic accuracy studies is the assumption that the clinical assessment standards are 100% sensitive and specific themselves. That is, that the tests for vision, hearing, musculoskeletal impairment, speech and cognition are indeed “gold standards” against which the CFM can be measured. The justification for selection of the five clinical assessments along with measures to ensure accuracy of the tests and to reduce classification bias [47] have been presented in detail elsewhere [21,22,23] and is summarised in Appendix B.

The five clinical assessments did not cover all the functioning constructs that are covered by the whole CFM (the CFM-13), specifically self-care, anxiety/worry, depression/sadness, behaviour and socialisation. We attempted to overcome this limitation by making interpretations based on IRR and simple proportions reported in different severity levels of the CFM-13. However, an outstanding recommendation for further research is for a diagnostic accuracy study which adequately covers these constructs.

A relatively high proportion of cases were from special schools (76.2%) due to the limited numbers of children with disabilities in mainstream schools. To achieve the required sample size across all five impairment groups, recruitment had to allow for this imbalance. Despite this, the target sample of 52 in each clinical impairment category was not reached for children with vision impairments (*n* = 35) and musculoskeletal impairments (*n* = 42). Future research should aim to rectify this sampling disparity and shortfall.

An important limitation relates to generalizing the findings to other populations. Of the parents/caregivers of the cases, 19% had attained a tertiary education, which is higher than the national average [48]. The level amongst controls was 15%, which is closer to average. This highlights potential differences related to parents of children in special schools, but importantly raises the question of difference between parents of children with disabilities in school compared to those who are out of school. Future research should include out-of-school children with disabilities, whose parents may respond differently to the CFM questions.

Another limitation is that 62.8% of cases were male compared to 49.0% of controls and the mean age of cases was 10.15 years compared to 9.71 years amongst controls. However, correlations between age, sex and the CFM questions were explored, and the impact of these variations appears to be negligible. Age had significant but negligible correlation with the domains learning (0.164), remembering (0.118) and depression (0.097). Sex had significant but negligible correlation with the domains speaking (0.092), learning (0.144), controlling behaviour (0.156), focusing attention (0.096) and making friends (0.097).

Finally, the authors acknowledge the limitations of categorizing IRR values into the classifications “excellent/good/fair/poor” because it is dependent on the purpose for which the test is to be used. For the purpose of this study however, the categories provide a convenient means of comparing individual domains and the overall CFM-13.

## 5. Conclusions

The UNICEF/WG Child Functioning Module is an important new instrument for disability disaggregation of datasets particularly considering the urgency to collect baseline information for the SDGs. When evaluated as a whole it achieved only a “fair” level of accuracy to identify children with disabilities in Fiji. This contrasts with earlier domain-specific findings which showed “good” to “excellent” accuracy for seeing, hearing, walking and speaking.

The choice of cut-off level and the mixture of severity of impairments reported across response categories are particular challenges for the CFM. Specifically, the response category “some difficulty” includes children with severe impairments as well as children with no impairments, with uneven results across disability domains. In the context of Fiji’s education system, children reported as having “some difficulty” can neither be ignored nor be assumed to have disability. There is no clear and strong cut-off response category for the overall CFM and the cut-off which performs best for individual functional domains is different from that for the overall module. While the CFM provides useful data for Fiji’s EMIS, the CFM is not accurate enough on its own for identifying children with disability for the purpose of determining funding eligibility.

We recommend that children with disabilities are identified using CFM plus additional data on learning and support needs and that verification visits are undertaken to confirm funding eligibility.

## Figures and Tables

**Table 1:** Demographic characteristics of the study sample.

*n* = 472, Unless Otherwise Stated		Cases (*n* = 231, 48.9%)		Controls (*n* = 241, 51.1%)	
		*n*	%	*n*	%
Gender	Male	145	62.8	118	49.0
	Female	86	37.2	123	51.0
Age (years)	5–7	43	18.6	52	21.6
	8–9	52	22.5	53	22.0
	10–11	42	18.2	59	24.5
	12–13	51	22.1	57	23.7
	14–15	43	18.6	20	8.3
Ethnicity	i-Taukei (Fijian)	141	61.0	159	66.0
	Indo-Fijian	75	32.5	78	32.4
	Other	15	6.5	4	1.7
Type of school	Special	176	76.2	56	23.2
	Mainstream primary	55	23.8	185	76.8
Parent/guardian respondent	Mother	130	56.3	144	59.8
	Father	44	19.0	61	25.3
	Other *	57	24.7	36	14.9
Highest level of education of parent	Primary	57	25.4	52	22.3
	Secondary	125	55.8	146	62.7
	Higher education	42	18.8	35	15.0
Area of Residence	Urban	63	27.3	44	18.3
	Peri-urban	112	48.5	68	28.2
	Rural	45	19.5	79	32.8
	Remote	11	4.8	50	20.7

* Other: grandparent, aunty, uncle, guardian.

**Table 2:** CFM domains, question wording and response categories; coded to indicate which group of domains was used in the various analyses in this paper.

Code Used in This Paper		Domain	CFM Question	Response Categories
CFM-13	CFM-7	Seeing	** Does (*child’s name)* have difficulty seeing?	(1) No difficulty (2) Some difficulty (3) A lot of difficulty (4) Cannot do at all
		Hearing	** Does (*child’s name)* have difficulty hearing sounds like peoples’ voices or music?	
		Walking	** Does (*child’s name)* have difficulty walking 100 metres on level ground? Does (child’s name) have difficulty walking 500 metres on level ground?	
		Speaking	When (child’s name) speaks does he/she have any difficulty being understood by: People inside this household People outside this household	
		Learning	Compared with children of the same age, does (*name*) have difficulty learning things?	
		Remembering	Compared with children of the same age, does (*name*) have difficulty remembering things?	
		Focusing attention	Does (*name*) have difficulty focusing on an activity that he/she enjoys doing?	
		Self-care	Does (*name*) have difficulty with self-care such as feeding or dressing him/herself?	
		Accepting changes to routine	Does (*name*) have difficulty accepting changes in his/her routine?	
		Making friends	Does (*name*) have difficulty making friends?	
		Anxiety/ worry	How often does (*name*) seem anxious, nervous or worried?	(1) Daily (2) Weekly (3) Monthly (4) A few times a year (5) Never
		Depression/sadness	How often does (*name*) seem sad or depressed?	
		Controlling behaviour	Compared with children of the same age, how much difficulty does (*name*) have controlling his/her behaviour?	(1) No difficulty (2) The same or less (3) More (4) A lot more

** The CFM includes questions to establish whether the child wears glasses, uses a hearing aid, or uses any equipment or receives assistance for walking. If the child does use the assistive device, the question for seeing is “When wearing his/her glasses, does *(name)* have difficulty seeing?” Similar questions are asked for hearing and walking. The CFM has separate questions for difficulty walking with and without equipment for children who need equipment. Analysis for this paper includes: difficulty walking for children who do not need equipment, plus those who require equipment but have difficulty walking without their equipment (this allows comparison with the Rapid Assessment of Musculoskeletal Impairment which tests function without equipment).

**Table 3:** Diagnostic accuracy of the Child Functioning Module (CFM-7); parent versus teacher responses, comparing two cut-off levels: “some difficulty” to “a lot of difficulty”.

Domain	AUC		Youden Index “some difficulty”		Youden Index “a lot of difficulty”	
	Parent	Teacher	Parent	Teacher	Parent	Teacher
Overall CFM-7	0.763	0.786	0.31	0.38	0.36	0.39
Seeing	0.848	0.823	0.69	0.61	0.13	0.35
Hearing	0.847	0.846	0.66	0.67	0.38	0.49
Walking	0.889	0.869	0.73	0.69	0.57	0.47
Speaking	0.975	0.909	0.88	0.70	0.75	0.60
Learning	0.774	0.822	0.51	0.60	0.21	0.27
Remembering	0.663	0.781	0.29	0.54	0.14	0.17
Focusing attention	0.623	0.686	0.24	0.37	0.05	0.10

**Table 4:** Cross-tabulation: Child Functioning Module results (CFM-7) by level of impairment.

CFM	Total *n* (%)		Impairment Level Based on Reference (Clinical) Assessments *, *n* (%)							
Difficulty in any CFM-7 Domain *	Parent, *n* = 472	Teacher, *n* = 392	None		Mild		Moderate		Severe	
			Parent	Teacher	Parent	Teacher	Parent	Teacher	Parent	Teacher
**No**	84 (17.8)	85 (21.7)	78 (33.9)	74 (43.8)	2 (10.5)	2 (11.8)	3 (4.8)	6 (10.9)	1 (0.6)	3 (2.0)
**Some**	212 (44.9)	154 (39.3)	109 (47.4)	66 (39.1)	8 (42.1)	10 (58.8)	33 (52.4)	26 (47.3)	62 (38.8)	52 (34.4)
**A lot**	122 (25.8)	104 (26.5)	41 (17.8)	27 (16.0)	9 (47.4)	5 (29.4)	25 (39.7)	19 (34.5)	47 (29.4)	53 (35.1)
**Cannot do**	54 (11.4)	49 (12.5)	2 (0.9)	2 (1.2)	0 (0.0)	0 (0.0)	2 (3.2)	4 (7.3)	50 (31.3)	43 (28.5)

* Child is recorded in the highest level of difficulty from any of the CFM-7 questions on seeing, hearing, walking, being understood when speaking, learning, remembering and focusing attention, and in the highest level of severity from any of the five reference standard assessments for vision, hearing, musculoskeletal, speech and cognitive impairment.

**Table 5:** Proportion endorsing each domain at the cut-off level “some difficulty” compared to “a lot of difficulty”, and inter-rater reliability between parents versus teachers.

Cut-Off Level	≥ Some Difficulty/Weekly *					≥ Lot of Difficulty/Daily *				
Respondent	Parent *n* (%)	Teacher *n* (%)	ICC	95% CI	Sig.	Parent *n* (%)	Teacher *n* (%)	ICC	95% CI	Sig.
Self-care	95 (20.1)	84 (21.6)	0.72	0.66–0.77	0.000	11 (2.3)	24 (6.2)	0.42	0.30–0.53	0.000
Feeling anxious *	117 (24.8)	103 (26.9)	0.26	0.10–0.40	0.002	42 (8.9)	51 (13.3)	0.09	−0.02–0.25	0.186
Feeling sad *	121 (25.7)	89 (23.4)	0.19	0.01–0.34	0.021	36 (7.6)	35 (9.2)	0.08	−0.03–0.25	0.211
Controlling behaviour ^Ω^	NA	NA	-	-	-	67 (14.2)	72 (18.8)	0.20	0.02–0.34	0.015
Accepting changes	232 (49.4)	153 (39.2)	0.14	−0.05–0.29	0.075	39 (8.3)	27 (6.9)	0.09	−0.12–0.25	0.190
Making friends	79 (16.8)	85 (21.9)	0.34	0.19–0.46	0.000	14 (3.0)	21 (5.4)	0.25	0.82–0.38	0.003

ICC = Intraclass correlation; ^Ω^ = more difficulty and a lot more difficulty.

**Table 6:** Frequencies of any impairment occurring amongst children reported as having a highest level of difficulty of at least “some difficulty” compared to at least “a lot of difficulty” on any question on the CFM (CFM-13), comparing parent and teacher responses.

CFM-13 (Highest Level of Difficulty on Any Question)	Impairment Level Based on Reference Standard (Clinical) Assessments, *n*(%)									
	Controls						Cases			
	*n*		No Impairment		Mild Impairment		Moderate Impairment		Severe Impairment	
	P	T	Parent *n*= 230	Teacher *n*= 169	Parent *n*= 19	Teacher *n*= 17	Parent *n*= 63	Teacher *n*= 55	Parent *n*= 160	Teacher *n*= 151
Some difficulty	189	117	113 (59.8)	62 (53.0)	7 (3.7)	6 (5.1)	25 (13.2) *(39.7)*	18 (15.4) *(33.3)*	44 (23.3) *(27.5)*	31 (26.5) *(20.5)*
≥ Lot of difficulty	231	198	70 (30.3)	40 (20.2)	11 (4.8)	9 (4.5)	35 (15.2)	32 (16.2)	115 (49.8)	117 (59.1)
Intraclass correlation, 95% confidence intervals, significance			ICC = 0.61 (95%CI 0.47–0.71, 0.000)		ICC = 0.85 (95%CI 0.58–0.94, 0.000)		ICC = 0.06 (95%CI −0.62–0.46, 0.408)		ICC = 0.55 (95%CI 0.38–0.68, 0.000)	

**Table 7:** Frequencies of five types of impairment occurring amongst children reported as having a highest level of difficulty of at least “some difficulty” compared to at least “a lot of difficulty” on any question on the CFM (CFM-13), comparing parent and teacher responses.

CFM-13 (Highest Level of Difficulty on Any Question)	Impairment Level Based on Reference Standard (Clinical) Assessments, *n*(%)									
	Controls						Cases			
	*n*		No Vision Impairment (≥6/9 ^¥^)		Mild VI (<6/9 ≥6/18 ^¥^)		Moderate VI (<6/18 ≥6/60 ^¥^)		Severe-Blind (<6/60 ^¥^)	
	P	T	Parent	Teacher	Parent	Teacher	Parent	Teacher	Parent	Teacher
Some difficulty	169	109	149 (88.2)	101 (92.7)	2 (1.2)	2 (1.8)	4 (2.4)	1 (0.9)	14 (8.3)	5 (4.6)
≥ Lot of difficulty	196	157	176 (89.8)	134 (85.4)	3 (1.5)	2 (1.3)	7 (3.6)	7 (4.5)	10 (5.1)	14 (8.9)
	*n*		No Hearing Impairment(<26 dBA)		Mild HI (26–40 dBA)		Moderate HI (41–60 dBA)		Severe-Profound HI (≥61 dBA)	
	P	T	Parent	Teacher	Parent	Teacher	Parent	Teacher	Parent	Teacher
Some difficulty	164	103	138 (84.1)	85 (82.5)	15 (9.1)	11 (10.7)	8 (4.9)	4 (3.9)	3 (1.8)	3 (2.9)
≥ Lot of difficulty	145	132	110 (66.3)	78 (59.1)	15 (9.0)	16 (12.1)	12 (7.2)	11 (8.3)	29 (17.5)	27 (20.5)
	*n*		No musculoskeletal impairment (MSI) ^		Mild MSI (5–24%) ^		Moderate MSI (25–49%) ^		Severe MSI (50–90%) ^	
	P	T	Parent	Teacher	Parent	Teacher	Parent	Teacher	Parent	Teacher
Some difficulty	175	111	169 (96.6)	105 (94.6)	3 (1.7)	2 (1.8)	3 (1.7)	3 (2.7)	0 (0.0)	1 (0.9)
≥ Lot of difficulty	208	185	172 (82.7)	152 (82.2)	6 (2.9)	6 (3.2)	11 (5.3)	11 (5.9)	19 (9.1)	16 (8.6)
	*n*		No speech impairment (4.0–5.0 ICS) ^₱^		Inconclusive speech function (2.5 < 4.0 ICS) ^₱^		Moderate speech impairment (1.8 < 2.5 ICS) ^₱^		Severe speech impairment (1.0 < 1.8 ICS) ^₱^	
	P	T	Parent	Teacher	Parent	Teacher	Parent	Teacher	Parent	Teacher
Some difficulty	185	114	141 (76.2)	72 (63.2)	38 (20.5)	31 (27.2)	4 (2.2)	3 (2.6)	2 (1.1)	8 (7.0)
≥ Lot of difficulty	226	194	71 (31.4)	71 (36.6)	90 (39.8)	68 (35.1)	17 (7.5)	16 (8.2)	48 (21.2)	39 (20.1)
	*n*		Average/better cognitive function		Low average cognitive function		Moderate cognitive Impairment		Severe cognitive impairment	
	P	T	Parent	Teacher	Parent	Teacher	Parent	Teacher	Parent	Teacher
Some difficulty	91	67	14 (15.4)	6 (9.0)	35 (38.5)	27 (40.3)	14 (15.4)	16 (23.9)	28 (30.8)	18 (26.9)
≥ Lot of difficulty	108	102	5 (4.6)	3 (2.9)	24 (22.2)	16 (15.7)	30 (27.8)	25 (24.5)	49 (45.4)	58 (56.9)

VI = Vision impairment; HI=Hearing impairment; MSI = Musculoskeletal impairment (mobility only); ^¥^ Visual Acuity of better eye; NPL—no perception of light; CF2m—counting fingers at 2metres. ^ Severity for the Rapid Assessment of Musculoskeletal Impairment was determined using the parameters for the percentage of function outlined in the International Classification of Functioning (ICF) [42]. Percentage loss of the musculoskeletal systems ability to function as a whole. ₱ Intelligibility in Context Scale—scores between 1.0–2.43 (detailed in [23]. For this paper, severe vision impairment and blindness are combined in one category and severe and profound hearing impairment are combined in one category. Results with these severities separately reported is available in [21].

## Supplementary Materials

The following are available online at https://www.mdpi.com/1660-4601/16/5/806/s1, Figure S1: Flowchart of participation, Table S1: Clinical characteristics of the study sample, Table S2: Extended data for Table 3—Diagnostic accuracy of the CFM-7 compared to five reference standard assessments, parent versus teacher responses, at different cut-off levels, Table S3: Extended data for Table 4—Cross-tabulation: Child Functioning Module results (CFM-7) by the results of the reference standard tests for vision, hearing, musculoskeletal, speech and cognition.

## Appendix A. Differences between the CFM Draft Version Used in this Study and the Final Version

(1) The draft version did not include reference to contact lenses, which is now in the final version, as shown in italics in the question: When wearing his/her glasses *or contact lenses*, does (name) have difficulty seeing?

(2) The word “focusing” has been replaced by “concentrating” in the final version.

“Does (*name*) have difficulty concentrating on an activity that he/she enjoys doing?”

(3) The question on difficulty controlling behaviour has been changed from the draft version used in this study:

“Compared with children of the same age, how much difficulty does (name) have controlling his/her behaviour?” with response options: No difficulty/The same or less/More/A lot more

To the final version: “Compared with children of the same age, does (name) have difficulty controlling his/her behaviour?” with response options: No difficulty/Some difficulty/A lot of difficulty/Cannot do at all.

(4) The word “very” has been inserted in the questions on anxiety and depression in the final version.

“How often does (*name*) seem very anxious, nervous or worried?”

“How often does (*name*) seem very sad or depressed?”

(5) The sequence of the last 8 questions has been changed in the final version.

In the draft version the CFM questions were in this sequence: learning, remembering, anxiety, depression, controlling behaviour, focusing attention, accepting changes in routine, and making friends.

In the final version the CFM questions are in this sequence: learning, remembering, concentrating (formerly focusing attention), accepting changes in routine, controlling behaviour, making friends, anxiety and depression.

## Appendix B. Description and Implementation of the Assessments

The reference standard (clinical) tests for this study were selected based on international standards for vision and hearing and well validated tools for speech, musculoskeletal impairment and cognitive impairment. The clinical team consisted of trained vision and hearing technicians and physiotherapists.

*Vision* assessment was performed with torchlight examination, visual acuity with Snellen chart, pinhole testing and refraction using a Topcon autorefractor. The following levels of vision impairment were included as cases: presenting visual acuity in the better eye <6/18 and ≥6/60 (moderate), <6/60 and ≥3/60 (severe) and <3/60 (blind) [21].

*Hearing* assessment was performed by observation with otoscope and air conduction audiometer. The pure-tone audiometry values for four frequencies in each ear, including 0.5, 1, 2 and 4 kHz, were averaged and the threshold level of the better ear was used to determine cut-off for cases. The following levels of hearing loss were included as cases: 41–60 dBA (moderate), 61–80 dBA (severe) and ≥81 dBA (profound). Greater than 30 or 31 dBA is commonly used as a criterion for hearing impairment in children [49,50], however >40 dBA was used in this study to identify children with clinically relevant hearing impairment due to the ambient noise levels in the assessment rooms in the schools. This is consistent with the extensive prior experience of the hearing assessors in Fiji and with other studies in developing countries [51,52]. Children found to have impacted wax or foreign bodies in the ear had this removed and were tested for hearing after removal.

*Musculoskeletal* assessment was undertaken using the Rapid Assessment of Musculoskeletal Impairment (RAMI) [30]. Through consultations with the Ministry of Health senior physiotherapist, it was established that there is no standard assessment used or validated for children of this age group for Fiji. Based on a literature review of assessment tools, the RAMI was deemed to be the best available method for establishing presence or absence of mobility impairments in this study setting [53]. The RAMI includes an initial set of five questions, such as, “Do you have any difficulty using your legs?”, with corresponding questions about duration indicating that it has lasted more than one month or is permanent. This is followed by observation of a series of gross and fine motor activities. In children where one or more of the five questions was answered positively, and one or more of the duration questions was “Yes”, and one or more of the observations indicated difficulty with the activities, children were assessed further for the extent of the effect on the musculoskeletal system. The RAMI does not consider functioning with equipment. Children identified on the RAMI to have impairment only affecting the upper limb were excluded for this analysis on walking difficulty. Children identified on the RAMI with structure impairment including “severe”, “moderate” and “mild” effect on the musculoskeletal system’s ability to function as a whole were identified as cases with mobility impairment [30].

*Speech* was assessed by administering the Intelligibility in Context Scale (ICS) [31] to parents. The ICS was selected as the tool to identify children with speech difficulties for several reasons: at time of data collection, there were no speech-language pathology services in Fiji and no speech assessment tools developed or validated in Fiji [54]. It can be administered by non-specialists. It can be used irrespective of language or number of languages spoken by the child [55,56], which is important in Fiji where many people are multilingual [57]. It assesses intelligibility and comprehensibility, which are comparable constructs to CFM questions on difficulty being understood when speaking. The ICS had already been rigorously translated into Fijian and Fiji-Hindi and has been widely used both with children with speech sound disorders [31,58] and with typically developing speech [32,58,59]. For our study, case definition for speech difficulties were ICS scores: 1.8 to <2.5 (moderate) and 1.0 to <1.8 (severe).

*Cognitive impairment* was assessed using the Cambridge Neuropsychological Test Automated Battery (CANTAB) [32] and cases included subjects with CANTAB Overall Impairment Scores of 3 (moderate) and 4–5 (severe). CANTAB, designed to be non-linguistic and culturally independent, has been validated with children to assess a range of cognitive functions [32,60,61,62] and has been used with children in a range of settings globally including where English is not the first language [63,64]. Five sub-tests, recommended by Cambridge Cognition to provide an overall assessment of cognitive function, were implemented in this order: Motor screening (MOT), Paired Associates Learning (PAL), Spatial Working Memory (SWM), Stockings of Cambridge (SOC) and Reaction Time (RTI).
