# Supplementary material for: The UNICEF/Washington Group Child Functioning Module—Accuracy, Inter-Rater Reliability and Cut-Off Level for Disability Disaggregation of Fiji’s Education Management Information System
Source: Int J Environ Res Public Health. 2019 Mar 5;16(5):806. doi: 10.3390/ijerph16050806 (PMC6427525; doi:10.3390/ijerph16050806)
Supplement: Supplementary file 1 [file ijerph-16-00806-s001.pdf]

International Journal of
Environmental Research and Public Health

MDPI

Supplementary Material

The UNICEF/Washington Group Child Functioning Module—Accuracy, Inter-Rater Reliability and Cut-Off Level for Disability Disaggregation of Fiji's Education Management Information System

Beth Sprunt *, Barbara McPake and Manjula Marella

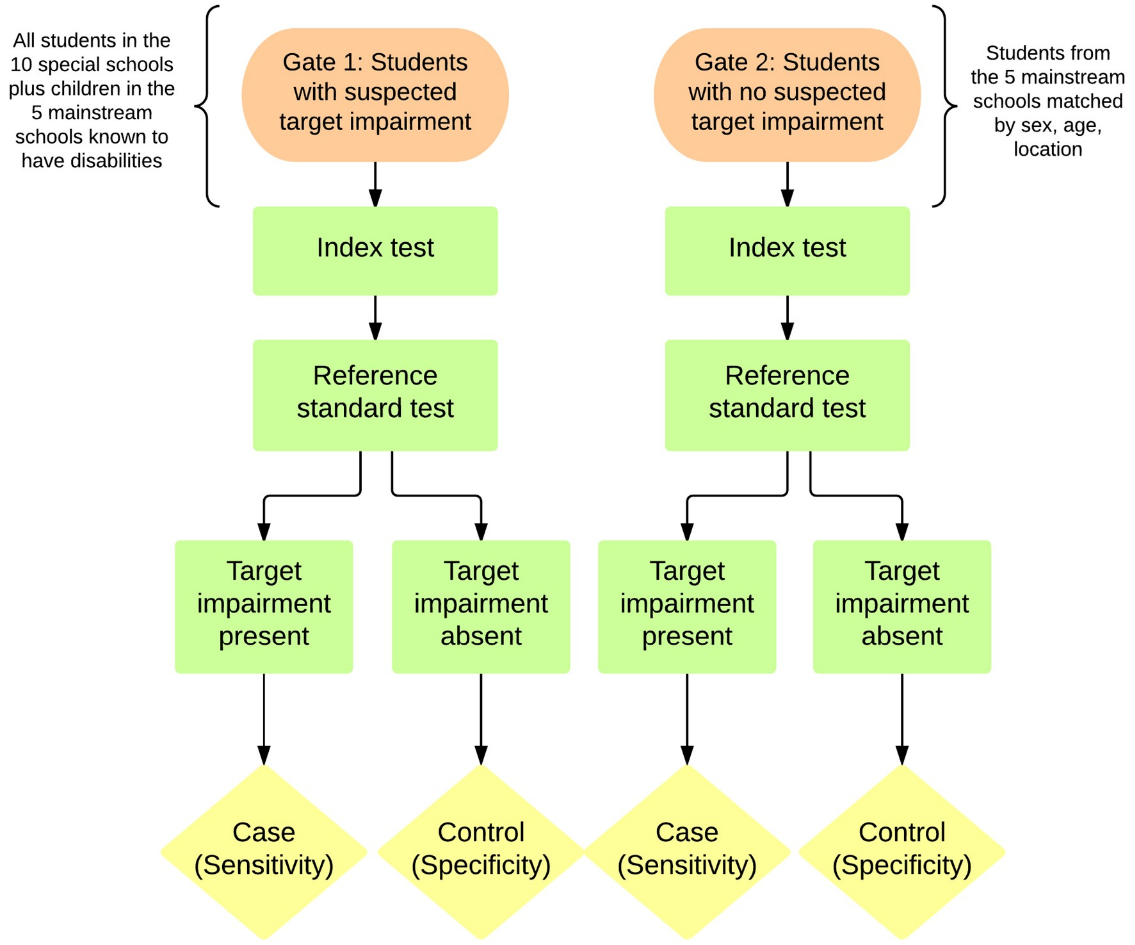

Figure S1. Flowchart of participation.

Table S1. Clinical characteristics of the study sample.

	Cases highlighted in grey	n	%
Vision impairment (n = 416)	None (≥6/9 ¥)	376	90.4
	Mild (<6/9 ≥6/18 ¥)	5	1.2
	Moderate (<6/18 ≥6/60 ¥)	11	2.6
	Severe (<6/60 ≥3/60 ¥)	2	0.5
	Blind (<3/60 ¥)	22	5.3
Hearing impairment (n = 381)	None (<26dBA)	298	78.2
	Mild (26-40dBA)	23	6.0
	Moderate (41-60dBA)	24	6.3
	Severe (61-80dBA)	8	2.1
	Profound (≥81dBA)	28	7.3
Musculoskeletal impairment (n = 435)^	None	393	90.3
	Mild (5–24%)	9	2.1
	Moderate (25–49%)	14	3.2
	Severe (50–90%)	19	4.4
Speech impairment (n = 462)	None (4.0-5.0 ICS score)	257	55.6
	Inconclusive speech function (2.5<4.0 ICS score)	134	29.0
	Moderate (<2.5 ≥1.8 ICS score)	21	4.5
	Severe (<1.8 ICS score)	50	10.8
Cognitive impairment (n = 225)	Average/better cognitive function	25	11.1
	Low average cognitive function	75	33.3
	Moderate impairment (Overall Impairment Score 3)	47	20.9
	Severe impairment (Overall Impairment Score 4 or 5)	78	34.7

¥ Visual Acuity of better eye; NPL – no perception of light; CF2m – counting fingers at 2metres.
^Severity for the Rapid Assessment of Musculoskeletal Impairment was determined using the parameters for the percentage of function outlined in the International Classification of Functioning (ICF). Percentage loss of the musculoskeletal systems ability to function as a whole. Participants categorised as 'mild' were included as cases based on detailed consideration of each assessment; diagnoses in the mild category included: club foot, head injury, epilepsy, limb pain limiting function, developmental delay, knock knees, and 'other neurological'. 5 of the 9 mild cases had a 2nd diagnosis.
Shaded area represents the numbers of cases according to study definitions.

Table S2. Extended data for Table 3 - Diagnostic accuracy of the CFM-7 compared to five reference standard assessments, parent versus teacher responses, at different cut-off levels.

Data on individual disability domains (sections in the table below the CFM-7 combined results) have been previously published and are reproduced here for comparability. Seeing, hearing and walking [21]; speaking [23]; learning, remembering and focusing attention [22].

Cut-off points	Sensitivity (Sn) (95% CI)	Specificity (Sp) (95% CI)	Youden Index (Se+Sp-1)	+LR (95% CI)	-LR (95% CI)
CFM-7 – combined results					
Parent	AUC 0.763, SE a: 0.021, 95%CI b (0.721, 0.804), p<0.0001, n=472, cases=231				
Some difficulty	0.98 (0.95, 0.99)	0.33 (0.27, 0.39)	0.31	1.46 (1.34, 1.60)	0.05 (0.02, 0.14)
A lot of difficulty	0.55 (0.49, 0.62)	0.80 (0.74, 0.85)	0.36**	2.78 (2.11, 3.68)	0.56 (0.48, 0.64)
Cannot do at all	0.23 (0.17, 0.29)	0.99 (0.96, 1.00)	0.21	18.08 (5.73, 57.09)	0.79 (0.73, 0.84)
Teacher	AUC 0.786, SE a: 0.023, 95%CI b (0.742, 0.831), p<0.0001, n=392, cases=212				
Some difficulty	0.96 (0.92, 0.98)	0.42 (0.35, 0.50)	0.38	1.66 (1.46, 1.88)	0.10 (0.05, 0.19)
A lot of difficulty	0.57 (0.50, 0.64)	0.82 (0.76, 0.87)	0.39**	3.21 (2.30, 4.49)	0.52 (0.45, 0.61)
Cannot do at all	0.22 (0.17, 0.29)	0.99 (0.96, 1.00)	0.21	19.95 (4.92, 81.00)	0.79 (0.73, 0.85)
Seeing					
Parent	AUC 0.848, SE a: 0.036, 95%CI b (0.809, 0.881), p<0.0001, n=409, cases=35				
Some difficulty	0.80 (0.63, 0.92)	0.89 (0.85, 0.92)	0.69**	7.12 (5.10, 9.90)	0.23 (0.10, 0.40)
A lot of difficulty	0.14 (0.04, 0.30)	0.99 (0.97, 1.00)	0.13	13.36 (3.80, 47.50)	0.87 (0.80, 1.00)
Cannot do at all	0.03 (0.00, 0.15)	1.00 (0.99, 1.00)	0.03	10.69 (0.70, 167.20)	0.97 (0.90, 1.00)
Teacher	AUC 0.823, SE a: 0.046, 95%CI b (0.779, 0.862), p<0.0001, n=340, cases=28				
Some difficulty	0.71 (0.51, 0.87)	0.90 (0.86, 0.93)	0.61**	7.43 (4.90, 11.2)	0.32 (0.20, 0.60)
A lot of difficulty	0.36 (0.19, 0.56)	0.99 (0.97, 1.00)	0.35	37.14 (10.80, 127.20)	0.65 (0.50, 0.90)
Cannot do at all	0.11 (0.02, 0.28)	1.00 (0.99, 1.00)	0.11	-	0.89 (0.80, 1.00)

Hearing					
Parent	AUC 0.847, SE [a]: 0.030, 95%CI [b] (0.806, 0.882), p<0.0001, n=378, cases=59				
Some difficulty	0.78 (0.65, 0.88)	0.88 (0.84, 0.91)	0.66**	6.38 (4.60, 8.80)	0.25 (0.20, 0.40)
A lot of difficulty	0.41 (0.28, 0.54)	0.97 (0.95, 0.99)	0.38	16.22 (7.70, 34.40)	0.61 (0.50, 0.80)
Cannot do at all	0.24 (0.14, 0.37)	1.00 (0.9, 1.00)	0.24	-	0.76 (0.70, 0.90)
Teacher	AUC 0.846, SE [a]: 0.032, 95%CI [b] (0.801, 0.885), p<0.0001, n=310, cases=54				
Some difficulty	0.72 (0.58, 0.84)	0.95 (0.92, 0.98)	0.67**	15.41 (8.70, 27.40)	0.29 (0.20, 0.40)
A lot of difficulty	0.50 (0.36, 0.64)	0.99 (0.97, 1.00)	0.49	64.00 (15.7, 261.1)	0.50 (0.40, 0.70)
Cannot do at all	0.19 (0.09, 0.31)	1.00 (0.98, 1.00)	0.19	47.41 (6.20, 362.6)	0.82 (0.70, 0.90)
Walking					
Parent	AUC 0.889, SE [a]: 0.031, 95%CI [b] (0.856, 0.917), p<0.0001, n=435, cases=42				
Some difficulty	0.83 (0.69, 0.93)	0.90 (0.87, 0.93)	0.73**	8.40 (6.10, 11.60)	0.19 (0.09, 0.40)
A lot of difficulty	0.60 (0.43, 0.74)	0.97 (0.95, 0.98)	0.57	19.49 (10.60, 35.90)	0.42 (0.30, 0.60)
Cannot do at all	0.38 (0.24, 0.54)	1.00 (0.99, 1.00)	0.38	149.71 (20.40, 1100.80)	0.62 (0.50, 0.80)
Teacher	AUC 0.869, SE [a]: 0.034, 95%CI [b] (0.830, 0.901), p<0.0001, n=368, cases=39				
Some difficulty	0.82 (0.67, 0.93)	0.87 (0.83, 0.90)	0.69**	6.14 (4.50, 8.40)	0.21 (0.10, 0.40)
A lot of difficulty	0.49 (0.32, 0.65)	0.98 (0.95, 0.99)	0.47	20.04 (9.40, 42.70)	0.53 (0.40, 0.70)
Cannot do at all	0.28 (0.15, 0.45)	1.00 (0.98, 1.00)	0.28	92.79 (12.3, 699.60)	0.72 (0.60, 0.90)
Speaking (combining variables on being understood by people inside and outside the house)					
Parent	AUC 0.975, SE: 0.00582 [a], CI (0.958, 0.992)[b], p<0.0001, n=328, cases=71				
Some difficulty	1.00 (0.95, 1.00)	0.88 (0.83, 0.92)	0.88**	8.32 (6.0, 11.6)	0.00
A lot of difficulty	0.76 (0.65, 0.85)	0.99 (0.97, 1.00)	0.75	65.41 (21.1, 203.0)	0.24 (0.2, 0.4)
Cannot do at all	0.45 (0.33, 0.57)	1.00 (0.98, 1.00)	0.45	116.28 (16.2, 836.3)	0.55 (0.4, 0.7)
Teacher	AUC 0.909, SE: 0.0198 [a], CI (0.874, 0.944)[b], p<0.0001, n=270, cases=67				
Some difficulty	0.94 (0.85, 0.98)	0.76 (0.69, 0.82)	0.70**	3.90 (3.0, 5.0)	0.08 (0.03, 0.2)
A lot of difficulty	0.63 (0.50, 0.74)	0.97 (0.94, 1.00)	0.60	21.21 (9.4, 47.7)	0.38 (0.3, 0.5)
Cannot do at all	0.16 (0.09, 0.28)	1.00 (0.98, 1.00)	0.16	-	0.84 (0.8, 0.9)
Learning					
Parent	AUC 0.774, SE[a]: 0.032, 95%CI [b] (0.712, 0.836), p<0.000, n=225, cases=125				
Some difficulty	0.78 (0.69, 0.84)	0.73 (0.63, 0.81)	0.51**	2.87 (2.05, 4.02)	0.31 (0.22, 0.43)
A lot of difficulty	0.24 (0.17, 0.33)	0.97 (0.91, 0.99)	0.21	8.0 (2.51, 25.45)	0.78 (0.71, 0.87)
Cannot do at all	0.02 (0.00, 0.06)	1.00 (0.95, 1.00)	0.02	Infinity (NA, Infinity)	0.98 (0.96, 10.1)
Teacher	AUC 0.822, SE[a]: 0.030, 95%CI[b] (0.763, 0.881), p<0.000, n=212, cases=120				
Some difficulty	0.88 (0.81, 0.93)	0.72 (0.61, 0.80)	0.60**	3.13 (2.24, 4.36)	0.16 (0.10, 0.27)
A lot of difficulty	0.32 (0.23, 0.41)	0.95 (0.87, 0.98)	0.27	5.83 (2.34, 14.23)	0.72 (0.64, 0.82)
Cannot do at all	0.03 (0.01, 0.09)	1.00 (0.95, 1.00)	0.03	Infinity (NA, Infinity)	0.97 (0.94, 1.00)
Remembering					
Parent	AUC 0.663, SE[a]: 0.036, 95%CI [b] (0.593, 0.733), p<0.000, n=225, cases=125				
Some difficulty	0.57 (0.48, 0.66)	0.72 (0.62, 0.80)	0.29**	2.03 (1.43, 2.88)	0.60 (0.48, 0.74)
A lot of difficulty	0.14 (0.08, 0.21)	1.00 (0.95, 1.00)	0.14	Infinity (NA, Infinity)	0.86 (0.81, 0.93)
Cannot do at all	0.01 (0.00, 0.05)	1.00 (0.95, 1.00)	0.01	Infinity (NA, Infinity)	0.99 (0.98, 1.01)
Teacher	AUC 0.781, SE[a]: 0.033, 95%CI [b] (0.716, 0.845), p<0.000, n=209, cases=1210				
Some difficulty	0.80 (0.72, 0.87)	0.74 (0.64, 0.83)	0.54**	3.10 (2.15, 4.45)	0.27 (0.19, 0.39)
A lot of difficulty	0.21 (0.14, 0.29)	0.96 (0.88, 0.99)	0.17	4.64 (1.67, 12.85)	0.83 (0.76, 0.91)
Cannot do at all	0.04 (0.02, 0.10)	1.00 (0.95, 1.00)	0.04	Infinity (NA, Infinity)	0.96 (0.92, 0.99)
Focusing Attention					
Parent	AUC 0.623, SE[a]: 0.037, 95%CI[b] (0.550, 0.696), p<0.002, n=224, cases=124				
Some difficulty	0.44 (0.36, 0.54)	0.80 (0.71, 0.87)	0.24**	2.21 (1.43, 3.44)	0.70 (0.59, 0.82)
A lot of difficulty	0.08 (0.04, 0.15)	0.97 (0.91, 0.99)	0.05	2.69 (0.76, 9.51)	0.95 (0.90, 1.00)
Cannot do at all	0.02 (0.00, 0.06)	1.00 (0.95, 1.00)	0.02	Infinity (NA, Infinity)	0.98 (0.96, 1.01)
Teacher	AUC 0.686, SE[a]: 0.037, 95%CI[b] (0.614, 0.758), p<0.000, N=210, cases=120				
Some difficulty	0.57 (0.47, 0.66)	0.80 (0.70, 0.87)	0.37**	2.83 (1.82, 4.41)	0.54 (0.44, 0.67)
A lot of difficulty	0.13 (0.07, 0.20)	0.97 (0.90, 0.99)	0.10	3.75 (1.12, 12.56)	0.91 (0.85, 0.97)
Cannot do at all	0.00 (0.00, 0.04)	1.00 (0.95, 1.00)	0.00	NA	1.00 (1.00, 1.00)

AUC: area under receiver operating characteristic curve; CI: confidence interval; SE: standard error; Positive likelihood ratio (+LR=Sn/(1-Sp); Negative likelihood ratio (-LR=(1-Sn)/Sp); **The highest Youden Index indicates the optimal cut-off point.

[a] DeLong et al., 1988; [b] Binomial exact

Case definition - child has impairment in at least one of: vision, hearing, musculoskeletal, speech or cognition.

CFM definition – the highest (most severe) response option selected in any one of the following questions: seeing, hearing, walking, speaking, learning, remembering, focusing attention. These questions are comparable to the impairment domains. CFM questions excluded from this ROC analysis include: self-care, anxiety, depression, controlling behaviour, accepting changes to routine and making friends.

Table S3. Extended data for Table 4 – Cross-tabulation: Child Functioning Module results (CFM-7) by the results of the reference standard tests for vision, hearing, musculoskeletal, speech and cognition.

CFM	Total n (%)		Impairment level based on reference standard (clinical) assessments, n (%)							
**Difficulty in any CFM-7 domain***	Parent, n=472	Teacher, n=392	None		Mild		Moderate		Severe	
			Parent	Teacher	Parent	Teacher	Parent	Teacher	Parent	Teacher
**No**	84 (17.8)	85 (21.7)	78 (92.9) *(33.9)*	74 (87.1) *(43.8)*	2 (2.4) *(10.5)*	2 (2.4) *(11.8)*	3 (3.6) *(4.8)*	6 (7.1) *(10.9)*	1 (1.2) *(0.6)*	3 (3.5) *(2.0)*
**Some**	212 (44.9)	154 (39.3)	109 (51.4) *(47.4)*	66 (42.9) *(39.1)*	8 (3.8) *(42.1)*	10 (6.5) *(58.8)*	33 (15.6) *(52.4)*	26 (16.9) *(47.3)*	62 (29.2) *(38.8)*	52 (33.8) *(34.4)*
**A lot**	122 (25.8)	104 (26.5)	41 (33.6) *(17.8)*	27 (26.0) *(16.0)*	9 (7.4) *(47.4)*	5 (4.8) *(29.4)*	25 (20.5) *(39.7)*	19 (18.3) *(34.5)*	47 (38.5) *(29.4)*	53 (51.0) *(35.1)*
**Cannot do**	54 (11.4)	49 (12.5)	2 (3.7) *(0.9)*	2 (4.1) *(1.2)*	0 (0.0) *(0.0)*	0 (0.0) *(0.0)*	2 (3.7) *(3.2)*	4 (8.2) *(7.3)*	50 (92.6) *(31.3)*	43 (87.8) *(28.5)*
**Difficulty Seeing**	Parent, n=409	Teacher, n=340	No vision impairment (VI) (≥6/9[¥])		Mild VI (<6/9 ≥6/18[¥])		Moderate VI (<6/18 ≥6/60[¥])		Severe-Blind (<6/60[¥])	
			Parent	Teacher	Parent	Teacher	Parent	Teacher	Parent	Teacher
**No**	339 (82.9)	290 (85.3)	330 (97.3) *(89.4)*	280 (96.6) *(91.2)*	2 (0.6) *(40.0)*	2 (0.7) *(40.0)*	6 (1.8) *(54.5)*	4 (1.4) *(50.0)*	1 (0.3) *(4.2)*	4 (1.4) *(20.0)*
**Some**	61 (14.9)	37 (10.9)	35 (57.4) *(9.5)*	25 (67.6) *(8.1)*	3 (4.9) *(60.0)*	2 (5.4) *(40.0)*	4 (6.6) *(36.4)*	3 (8.1) *(37.5)*	19 (31.1) *(79.2)*	7 (18.9) *(35.0)*
**A lot**	7 (1.7)	10 (2.9)	3 (42.9) *(0.8)*	2 (20.0) *(0.7)*	0 (0.0) *(0.0)*	1 (10.0) *(20.0)*	1 (14.3) *(9.1)*	1 (10.0) *(12.5)*	3 (42.9) *(12.5)*	6 (60.0) *(30.0)*
**Cannot do**	2 (0.5)	3 (0.9)	1 (50.0) *(0.3)*	0 (0.0) *(0.0)*	0 (0.0) *(0.0)*	0 (0.0) *(0.0)*	0 (0.0) *(0.0)*	0 (0.0) *(0.0)*	1 (50.0) *(4.2)*	3 (100.0) *(15.0)*
**Difficulty Hearing**	Parent, n=378	Teacher, n=301	No hearing impairment (HI) (<26dBA)		Mild HI (26-40dBA)h		Moderate HI (41-60dBA)		Severe-Profound HI (≥61dBA)	
			Parent	Teacher	Parent	Teacher	Parent	Teacher	Parent	Teacher
**No**	293 (77.5)	251 (83.4)	263 (89.8) *(88.9)*	224 (89.2) *(95.7)*	17 (5.8) *(73.9)*	20 (8.0) *(90.9)*	9 (3.1) *(39.1)*	5 (2.0) *(33.3)*	4 (1.4) *(11.1)*	2 (0.8) *(6.7)*
**Some**	53 (14.0)	21 (7.0)	26 (49.1) *(8.8)*	9 (42.9) *(3.8)*	5 (9.4) *(21.7)*	1 (4.8) *(4.5)*	10 (18.9) *(43.5)*	4 (19.0) *(26.7)*	12 (22.6) *(33.3)*	7 (33.3) *(23.3)*
**A lot**	18 (4.8)	18 (6.0)	7 (38.9) *(2.4)*	0 (0.0) *(0.0)*	1 (5.6) *(4.3)*	1 (5.6) *(4.5)*	3 (16.7) *(13.0)*	2 (11.1) *(13.3)*	7 (38.9) *(19.4)*	15 (83.3) *(50.0)*
**Cannot do**	14 (3.7)	11 (3.7)	0 (0.0) *(0.0)*	1 (9.1) *(0.4)*	0 (0.0) *(0.0)*	0 (0.0) *(0.0)*	1 (7.1) *(4.3)*	4 (36.4) *(26.7)*	13 (92.9) *(36.1)*	6 (54.5) *(20.0)*
**Difficulty Walking[#]**	Parent, n=435	Teacher, n=368	No musculoskeletal impairment (MSI)		Mild MSI (5–24%)		Moderate MSI (25–49%)		Severe MSI (50–90%)	
			Parent	Teacher	Parent	Teacher	Parent	Teacher	Parent	Teacher
**No**	361 (83.0)	292 (79.3)	354 (98.1) *(90.1)*	285 (97.6) *(86.6)*	5 (1.4) *(55.5)*	4 (1.4) *(50.0)*	2 (0.6) *(14.3)*	3 (1.0) *(21.4)*	0 (0.0) *(0.0)*	1 (0.0) *(5.6)*
**Some**	37 (8.5)	49 (13.3)	27 (73.0) *(6.9)*	36 (73.5) *(10.9)*	2 (5.4) *(22.2)*	4 (8.2) *(50.0)*	8 (21.6) *(57.1)*	8 (16.3) *(57.1)*	0 (0.0) *(0.0)*	1 (2.0) *(5.6)*
**A lot**	20 (4.6)	15 (4.1)	11 (55.0) *(2.8)*	7 (46.7) *(2.1)*	2 (10.0) *(22.2)*	0 (0.0) *(0.0)*	3 (15.0) *(21.4)*	3 (20.0) *(21.4)*	4 (20.0) *(21.0)*	5 (33.3) *(27.8)*
**Cannot do**	17 (3.9)	12 (3.3)	1 (5.9) *(0.3)*	1 (8.3) *(0.3)*	0 (0.0) *(0.0)*	0 (0.0) *(0.0)*	1 (5.9) *(7.1)*	0 (0.0) *(0.0)*	15(88.2) *(79.0)*	11 (91.7) *(61.1)*
**Difficulty being understood by people inside**	Parent[£] n=462	Teacher¥, n=387	No speech impairment (4.0-5.0 ICS)[₱]		Inconclusive speech function (2.5<4.0 ICS)[₱]		Moderate speech impairment (1.8<2.5 ICS)[₱]		Severe speech impairment (1.0<1.8 ICS)[₱]	
			Parent	Teacher	Parent	Teacher	Parent	Teacher	Parent	Teacher
**No**	303 (65.6)	241 (62.3)	239 (78.9)	167 (68.9) *(81.1)*	60 (19.8)	57 (23.9) *(50.0)*	3 (0.99) *(14.3)*	3 (1.2) *(15.8)*	1 (0.3) *(2.0)*	14 (5.8) *(29.2)*

			(93.0)		*(44.8)*					
**Some**	96 (20.8)	94 (24.3)	17 (17.7) *(6.6)*	35 (37.2) *(17.0)*	59 (61.5) *(44.0)*	38 (40.4) *(33.3)*	14 (14.58) *(66.7)*	7 (7.5) *(36.8)*	6 (6.3) *(12.0)*	14 (14.9) *(29.2)*
**A lot**	31 (6.7)	38 (9.8)	1 (3.2) *(0.4)*	4 (10.5) *(1.9)*	13 (41.9) *(9.7)*	15 (39.5) *(13.2)*	3 (9.68) *(14.3)*	6 (15.8) *(31.6)*	14 (45.2) *(28.0)*	13 (34.2) *(27.1)*
**Cannot do**	32 (6.9)	14 (3.6)	0 (0.0) *(0.0)*	0 (0.0) *(0.0)*	2 (6.3) *(1.5)*	4 (28.6) *(3.5)*	1 (3.13) *(4.8)*	3 (21.4) *(15.8)*	29 (90.6) *(58.0)*	7 (50.0) *(14.6)*
**Difficulty being understood by people outside**	Parent*, n=462	Teacher¥, n=384	No speech impairment (4.0-5.0 ICS)₱		Inconclusive speech function (2.5<4.0 ICS)₱		Moderate speech impairment (1.8<2.5 ICS)₱		Severe speech impairment (1.0<1.8 ICS)₱	
			Parent	Teacher	Parent	Teacher	Parent	Teacher	Parent	Teacher
**No**	264 (57.1)	214 (55.7)	229 (86.7) *(89.1)*	159 (74.3) *(78.3)*	35 (13.3) *(26.1)*	51 (23.8) *(44.7)*	0 (0.0) *(0.0)*	1 (0.5) *(5.3)*	0 (0.0) *(0.0)*	3 (1.4) *(6.3)*
**Some**	115 (24.9)	100 (26.0)	26 (22.6) *(10.0)*	40 (40.0) *(19.7)*	71 (61.7) *(53.0)*	38 (38.0) *(33.3)*	11 (9.3) *(52.3)*	9 (9.0) *(47.4)*	7 (6.1) *(14.0)*	13 (13.0) *(27.1)*
**A lot**	50 (10.8)	57 (14.8)	2 (4.0) *(0.8)*	4 (7.0) *(2.0)*	26 (52.0) *(19.4)*	21 (36.8) *(18.4)*	7 (14.0) *(33.3)*	6 (10.5) *(31.6)*	15 (30.0) *(30.0)*	26 (45.6) *(54.2)*
**Cannot do**	33 (7.1)	13 (3.4)	0 (0.0) *(0.0)*	0 (0.0) *(0.0)*	2 (6.1) *(1.5)*	4 (30.8) *(3.5)*	3 (9.1) *(14.3)*	3 (23.1) *(15.8)*	28 (84.5) *(56.0)*	6 (18.2) *(12.5)*
**Difficulty Learning**	Parent, n=225	Teacher, n=212	Average/better cognitive function@		Low average cognitive function@		Moderate cognitive Impairment@		Severe cognitive impairment@	
			Parent	Teacher	Parent	Teacher	Parent	Teacher	Parent	Teacher
**No**	101 (44.9)	80 (37.7)	20 (19.8) *(80.0)*	17 (21.3) *(73.9)*	53 (52.5) *(70.7)*	49 (61.3) *(71.0)*	9 (8.9) *(19.1)*	5 (6.3) *(11.4)*	19 (18.8) *(24.4)*	9 (11.3) *(9.9)*
**Some**	91 (40.4)	89 (42.0)	4 (4.4) *(16.0)*	4 (4.5) *(17.4)*	20 (22.0) *(26.7)*	17 (19.1) *(24.6)*	27 (29.7) *(57.4)*	27 (30.3) *(61.4)*	40 (44.0) *(51.3)*	41 (46.1) *(45.1)*
**A lot**	31 (13.8)	54 (25.5)	1 (3.2) *(4.0)*	2 (5.1) *(8.7)*	2 (6.5) *(2.7)*	3 (7.7) *(4.3)*	11 (35.5) *(23.4)*	10 (25.6) *(22.7)*	17 (54.8) *(21.8)*	39 (72.2) *(42.9)*
**Cannot do**	2 (0.89)	4 (1.9)	0 (0.0) *(0.0)*	0 (0.0) *(0.0)*	0 (0.0) *(0.0)*	0 (0.0) *(0.0)*	0 (0.0) *(0.0)*	2 (50.0) *(4.5)*	2 (100.0) *(2.6)*	2 (50.0) *(2.2)*
**Difficulty Remembering**	Parent, n=225	Teacher, n=209	Parent	Teacher	Parent	Teacher	Parent	Teacher	Parent	Teacher
**No**	126 (56.0)	90 (43.1)	21 (16.7) *(84.0)*	17 (18.9) *(73.9)*	51 (40.5) *(68.0)*	49 (54.4) *(74.2)*	20 (15.9) *(42.6)*	8 (8.9) *(18.2)*	34 (27.0) *(43.6)*	16 (17.8) *(21.1)*
**Some**	82 (36.4)	90 (43.1)	4 (4.9) *(16.0)*	5 (5.6) *(21.7)*	24 (29.3) *(32.0)*	14 (15.6) *(21.2)*	20 (24.4) *(42.6)*	30 (33.3) *(68.2)*	34 (41.5) *(43.6)*	41 (45.6) *(53.9)*
**A lot**	16 (7.1)	24 (11.5)	0 (0.0) *(0.0)*	1 (4.2) *(4.3)*	0 (0.0) *(0.0)*	3 (12.5) *(4.5)*	7 (43.8) *(14.9)*	5 (20.8) *(11.4)*	9 (56.3) *(11.5)*	15 (62.5) *(19.7)*
**Cannot do**	1 (0.4)	5 (2.4)	0 (0.0) *(0.0)*	0 (0.0) *(0.0)*	0 (0.0) *(0.0)*	0 (0.0) *(0.0)*	0 (0.0) *(0.0)*	1 (20.0) *(2.3)*	1 (100.0) *(1.3)*	4 (80.0) *(5.3)*
**Difficulty Focusing**	Parent, n=224	Teacher, n=210	Parent	Teacher	Parent	Teacher	Parent	Teacher	Parent	Teacher
**No**	148 (66.4)	124 (59.0)	20 (13.4) *(80.0)*	20 (16.1) *(87.0)*	60 (40.3) *(80.0)*	52 (41.9) *(77.6)*	26 (17.4) *(55.3)*	28 (22.6) *(63.6)*	43 (28.9) *(55.8)*	24 (19.4) *(31.6)*
**Some**	63 (28.3)	68 (32.4)	4 (6.5) *(16.0)*	1 (1.5) *(4.3)*	13 (21.0) *(17.3)*	14 (20.6) *(20.9)*	16 (25.8) *(34.0)*	13 (19.1) *(29.5)*	29 (46.8) *(37.7)*	40 (58.8) *(52.6)*
**A lot**	10 (4.5)	18 (8.6)	1 (9.1) *(4.0)*	2 (11.1) *(8.7)*	2 (18.2) *(2.7)*	1 (5.6) *(1.5)*	5 (45.5) *(10.6)*	3 (16.7) *(6.8)*	3 (27.3) *(3.9)*	12 (66.7) *(15.8)*
**Cannot do**	2 (0.9)	0 (0.0)	0 (0.0) *(0.0)*	0 (0.0) *(0.0)*	0 (0.0) *(0.0)*	0 (0.0) *(0.0)*	0 (0.0) *(0.0)*	0 (0.0) *(0.0)*	2 (100.0) *(2.6)*	0 (0.0) *(0.0)*

*Child is recorded in the highest level of difficulty from any of the CFM-7 questions on seeing, hearing, walking, being understood when speaking, learning, remembering and focusing attention, and in the highest level of severity from any of the five reference standard assessments for vision, hearing, musculoskeletal, speech and cognitive impairment.
VI=Vision impairment; HI=Hearing impairment; MSI=Musculoskeletal impairment (mobility only);
¥ Visual Acuity of better eye; NPL – no perception of light; CF2m – counting fingers at 2metres.
^Severity for the Rapid Assessment of Musculoskeletal Impairment was determined using the parameters for the percentage of function outlined in the International Classification of Functioning (ICF)(43). Percentage loss of the musculoskeletal systems ability to function as a whole.

includes: difficulty walking for children who do not need equipment, plus those who require equipment but have difficulty walking without their equipment (this allows comparison with the Rapid Assessment of Musculoskeletal Impairment which tests function without equipment)
£ Parent: When speaking, level of difficulty being understood by people: inside the household / outside the household
¥ Teacher: When speaking, level of difficulty being understood by people: inside the classroom / outside the classroom
₱ Intelligibility in Context Scale - scores between 1.0-2.43, detailed in (23).
For this paper, severe vision impairment and blindness are combined in one category and severe and profound hearing impairment are combined in one category. Results with these severities separately reported is available in (21).

© 2019 by the authors. Submitted for possible open access publication under the terms and conditions of the Creative Commons Attribution (CC BY) license (http://creativecommons.org/licenses/by/4.0/).
